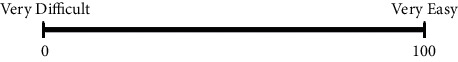# Aesthetic Parameters and Patient-Perspective Assessment Tools for Maxillary Anterior Single Implants

**DOI:** 10.1155/2021/6684028

**Published:** 2021-02-17

**Authors:** Kelvin I. Afrashtehfar, Mansour K. A. Assery, S. Ross Bryant

**Affiliations:** ^1^Division of Restorative Dental Sciences, Department of Clinical Sciences, College of Dentistry, Ajman University, 346 Ajman, UAE; ^2^Department of Reconstructive Dentistry and Gerodontology, School of Dental Medicine, Faculty of Medicine, University of Bern, 3010 Berne CH, Switzerland; ^3^Vicerrectorado de Investigación-EIDUCAM, Universidad Católica San Antonio de Murcia (UCAM), 30107 Murcia, Spain; ^4^Department of Oral Surgery and Stomatology, School of Dental Medicine, Faculty of Medicine, University of Bern, 3010 Berne CH, Switzerland; ^5^Department of Restorative Dentistry, Edinburgh Dental Institute, College of Medicine and Veterinary, University of Edinburgh, Edinburgh EH3 9HA, UK; ^6^Centre of Medical and Bio-allied Health Sciences Research (CMBHSR), Ajman University, Dubai, UAE; ^7^Department of Prosthodontics, College of Dentistry, Riyadh Elm University, Riyadh 12611, Saudi Arabia; ^8^Postgraduate Studies and Scientific Research, Riyadh Elm University, Riyadh 12611, Saudi Arabia; ^9^Department of Oral Health Sciences, Faculty of Dentistry, University of British Columbia, Vancouver BC V6T 1Z3, Canada; ^10^Division of Prosthodontics and Dental Geriatrics, Faculty of Dentistry, University of British Columbia, Vancouver BC V6T 1Z3, Canada

## Abstract

**Background:**

This review aimed to concisely describe the current aesthetic objective indices for a single-implant maxillary anterior crown. The secondary aim was to propose introducing a unified, standardized questionnaire for adequately collecting patient-reported outcome measures (PROMs) in implant dentistry.

**Materials and Methods:**

A literature review was conducted using both EMBASE/Ovid and MEDLINE/PubMed databases by combining keywords and Emtree/Mesh terms related to “Esthetics,” “Self-Assessment or Surveys and Questionnaires,” and “Single-Tooth Dental Implants.”

**Results:**

The most meaningful aesthetic objective indices for single implants in the literature are the Pink Esthetic Score (PES), the Papilla Presence Index (PPI), Peri‐Implant and Crown Index (PICI), PES/White Esthetic Score (PES/WES), the Implant Crown Aesthetic Index (ICAI), and a modified version of the ICAI (mod-ICAI) index. Clearly, PES/WES is still the most widely accepted tool. It is encouraging to observe that there is an increasing tendency in recent years to report PROMs more frequently in the implant dentistry literature. We proposed the implementation of a unified, standardized questionnaire using a self-administered visual analogue scale (VAS) scoring system, which evaluates overall satisfaction, comfort, tooth appearance, gingival appearance, function, and hygiene complexity. This tool should be validated in the oral implantology research context for its regular implementation or further development.

**Conclusions:**

Conducting qualitative studies among dental implant patients who received few implants or single-tooth implant reconstructions in the aesthetic zone may help dental researchers understand better how to efficiently develop and validate a quantitative instrument. This standard tool would reduce heterogeneity bias by providing comparable data between studies.

## 1. Introduction

Single missing maxillary teeth in the aesthetic zone (i.e., tooth sites that are mostly visible in the smile) are increasingly managed with dental implants, especially in cases where the adjacent teeth are relatively free of disease or damage.

### 1.1. Epidemiology of Missing Maxillary Anterior Teeth

Although the prevalence of tooth loss has been decreasing in recent decades [1], up to one-quarter of adults in Western countries are missing at least one anterior tooth [2, 3]. The aetiology of single missing permanent teeth in the aesthetic zone stems from either developmental hypodontia or acquired tooth loss. Hypodontia (i.e., tooth agenesis) is the most common developmental abnormality in humans [4], including those caused by both environmental and genetic factors [5–7]. Omitting third molars, the prevalence of hypodontia in the permanent dentition is reported to be up to 6.9% [7]. Additionally, maxillary lateral incisors are some of the most prevalent congenitally missing permanent teeth [8, 9].

Acquired loss of anterior teeth at a young age is most frequently due to dental trauma, but over the adult lifespan, the aetiology will encompass broader, multifactorial origins, often including dental caries, as well as periodontal disease and less common causes [10–13], such as persisting oral habits and neoplasia [14]. The estimated prevalence of anterior dental trauma between the ages of 6 and 17 ranged widely across several studies from 6.4% to 37.9% [15].

### 1.2. Treatment for a Single-Tooth Edentulous Space in the Anterior Maxillary Zone

The traditional treatment for a single-tooth edentulous space in the anterior maxillary region has been a conventional three-unit or cantilever fixed dental prosthesis (FDP) [16, 17]. Two significant shortcomings of these alternatives are the need for significant tooth reduction of the abutments and an increased risk of dental caries, the most common cause of subsequent prosthesis failure [18]. Furthermore, subgingival FDP margins are often required in visible regions of the mouth, but these are associated with increased chronic gingival inflammation, leaving the patient at risk of more serious periodontal disease.

All these can be avoided if an implant is utilized to replace the missing tooth, especially when the teeth adjacent to the edentulous zone are sound [19]. It is well-established that single-tooth implants have favourable long-term survival rates [20]. Nevertheless, it is challenging to replace a single missing tooth in the aesthetic zone with an implant since hard and soft tissue resorption defects are often present [21–24]. In one study, out of a total of 2,381 dental implants placed at a university clinic, 492 (20.8%) were placed in the anterior maxilla [25]. Frequent adjunct treatments to optimize the position and aesthetic results of such implants are bone grafting and soft tissue surgery. However, compromises to the final position and appearance often linger [21, 22, 26–33]. Patient-reported outcomes measures (PROMs) in dental medicine have been described as the fundamental “subjective” reports of patients' own perceptions of their oral health status and its impact on their daily life, including satisfaction with oral health status and other nonclinical assessments. However, PROMs have been underreported in almost all areas of dental medicine, and single-tooth implant treatment in the aesthetic zone is not the exception.

There is a need for reporting patient-reported outcome measures (PROMs) of single-tooth edentulous spaces in the anterior maxillary zone managed with a dental implant supporting a fixed reconstruction. Thus, this review aimed to concisely describe the current aesthetic objective indices for a single-implant maxillary anterior crown. The secondary aim was to propose introducing a unified, standardized questionnaire for adequately collecting PROMs in implant dentistry.

## 2. Materials and Methods

A narrative review approach was used for fulfilling the objectives of the present study. An electronic search was conducted aided by Embase/Ovid and MEDLINE/PubMed databases by combining keywords and Emtree/MeSH terms related to “Esthetics,” “Self-Assessment or Surveys and Questionnaires,” and “Single-Tooth Dental Implants.” The search that supported the literature review was carried out up to July 12, 2020. This was complemented by manual searching the references of relevant studies. Forty-three studies met the selection criteria; however, not all studies were considered as there was duplication among some of the secondary sources. Mostly, review studies, clinical trials, and cases and controls were included. No meta-analyses were found.

## 3. Results

The present narrative review provides an insight into the most meaningful aesthetic objective indices for single implants in the dental literature.

### 3.1. Objective and Subjective Outcomes of Maxillary Single-Implant Anterior Teeth

Impaired appearance is the most apparent reason individuals seek to restore missing anterior teeth. Nonetheless, the primary focus of early literature on maxillary anterior implant outcomes was based on survival parameters, with a lack of information regarding aesthetically relevant parameters [34–36].

### 3.2. There Is a Need for Reporting Patient-Reported Outcome Measures (PROMs) in Dental Medicine

Aesthetic outcome parameters have evolved to include both subjective (patient-mediated) and objective (dentist-mediated) quantitative outcomes [37–39]. In the process, researchers established that patient satisfaction was noticeably underreported in the early implant literature [36]. Subjective evaluation can be carried out using patient perceptions of the aesthetic outcome measured with specific questionnaires in which patients express their degree of satisfaction or dissatisfaction [40, 41]. Such patient-reported outcome measures (PROMs) have the purpose of integrating patients' opinions, which offer valuable additional information beyond clinical outcome parameters [42, 43]. The most popular formats for these PROMs are the Likert-type scale and the visual analogue scale (VAS) and, less commonly, a dichotomous coding system [28, 42].

Recent studies are now even more detailed on aesthetic outcomes than earlier reports of patient-based subjective satisfaction scores [37]. It has been pointed out that early reports on PROMs in implant dentistry focused on general patient satisfaction, which may not serve to adequately assess the range of impacts of implants on treatment outcomes as perceived by patients [44]. Thus, researchers have recommended adding more PROM-related detailed questions to give insight into a broader range of aspects that might affect patient satisfaction with implant prostheses [44].

### 3.3. Current Aesthetic Objective Indices for a Single-Implant Maxillary Anterior Crown

Several objective indices have been developed to assess clinician-mediated aesthetic outcomes for single-tooth implant restorations in the aesthetic zone [39, 45], including the Pink Esthetic Score (PES) [46], the Papilla Presence Index (PPI) [47], Peri‐Implant and Crown Index (PICI) [45], PES/White Esthetic Score (PES/WES) [34], the Implant Crown Aesthetic Index (ICAI) [48], and a modified version of the ICAI (mod-ICAI) index [49] (Table 1).

While it is not in the scope of the present study to discuss in detail these aesthetic indices, a recent systematic review of studies using them identified unexplained variability across the studies in the correlations reported between subjective (i.e., patient-based) questionnaires and objective (i.e., clinician-mediated) assessments [37]. Among these studies, the subjective evaluation method was mostly conducted via VAS (eight out of eleven studies) and the remaining (three out of eleven studies) by a Likert-type scale (5- or 6-point rating). Overall, patients' subjective scores were either significantly higher when compared with clinicians' objective scores, or no significant correlation was found between these two groups of evaluators [37].

#### 3.3.1. Example of a PES/WES Evaluation

**Figure 1:**
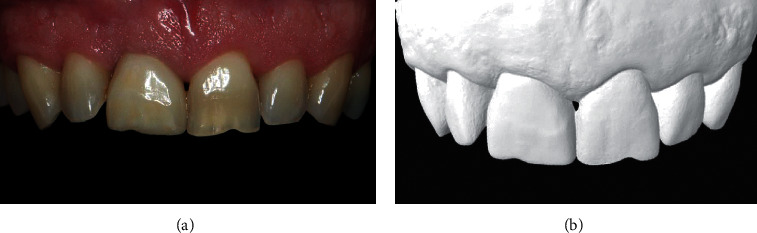
Dental records obtained from a male patient who had the right central incisor restored with a single implant-supported crown. (a) Maxillary anterior region with retracted cheeks and lips. (b) Digital image generated from the intraoral scanner.

This example displays a standardized intraoral front photograph with cheeks and lips retracted (Figure 1(a)) and a maxillary virtual cast model (i.e., Standard Tessellation Language (STL) file in Preview app version 7.0 (Apple Inc., Cupertino, CA, USA); Figure 1(b)) appraised by a validated objective aesthetic index such as the PES/WES [34, 50]. Two experienced clinician researchers, calibrated for aesthetic analyses, independently evaluated these bidimensional and tridimensional objects with score sheets (Table 2). A score of ≥6 (out of a maximum of 10) for either PES or WES and ≥12 (out of a maximum of 20) for PES/WES combined were generally considered satisfactory. For example, the average score from both evaluators obtained from the single clinical scenario displayed in Figure 1 was 8/10, 8/10, and 16/20 for PES, WES, and PES/WES, respectively. Therefore, the three accumulated scores (i.e., PES, WES, and PES/WES) of the displayed clinical scenario were considered satisfactory.

## 4. Discussion

This review aimed to concisely acknowledge the current aesthetic objective indices for a single-implant maxillary anterior crown. A secondary aim was to propose introducing a unified, standardized questionnaire for adequately collecting PROMs in implant dentistry.

### 4.1. Potential Factors Influencing Patient Satisfaction with Maxillary Anterior Single-Tooth Implants

Naturally, patient satisfaction with maxillary anterior single-tooth implants is likely influenced by a range of additional outcomes, beyond aesthetics, broadly related to function, including maintenance and complications, as well as other personal and environmental influences such as body image, dentist-patient relationship, patients' expectations, and financial restrictions. Among these, personal influence is the importance that patients may place on dentists successfully re-establishing a comfortable oral function with stable dental occlusion [51–55].

Understanding patients' functional experiences, perhaps most notably involving chewing and speech, is useful for discussing realistic functional outcomes with patients relative to their expectations. For example, it is known that speech difficulties may be encountered, especially initially, with the installation of maxillary implant-supported fixed dental prostheses (ISFDPs) [56, 57].

Given the difficulty in parsing out the influence of aesthetics on patient satisfaction, having a more thorough explanation of these other potential factors from a patient perspective would likely be useful in further understanding patient satisfaction with maxillary anterior single-tooth implants. It would also be interesting to know about what would seem to likely be a substantial impact on the social life and self-perception of patients with a single implant in the aesthetic zone, although this has not been the focus of prior investigation.

#### 4.1.1. Influence of Maintenance and Adverse Events

There is increasing information on the impact of maintenance care needed for dental implants, as well as the impact of potential technical complications over time [58–67]. For example, two qualitative studies have reported negative subjective experiences in some implant patients in terms of not being able to cleanse their ISFDPs properly [56, 57]. Yet little is known regarding how satisfied patients are with the verbal or written instructions provided on maintaining single-tooth implants [68].

Gathering further information on patient experiences with maintenance may, therefore, help to develop strategies to improve both satisfaction and compliance with maintenance and preventive recommendations for maxillary anterior single-tooth implants. On the issue of the impact of complications, single-implant crowns are associated with an increased incidence of technical adverse events (i.e., ceramic fractures or chipping of the veneer material, abutment or screw loosening, and loss of retention) compared to traditional tooth-borne crowns and splinted-implant crowns [69–71]. However, the survival of single-implant crowns in the anterior region was higher than the three-unit FDP alternative in a 15-year follow-up study [72].

Although it is not a specific goal of the present review to include data on the prevalence and impact of complications, it is important to acknowledge their possibility since single-tooth implant patients' perspectives may be influenced by complications experienced after treatment delivery. Interestingly, one qualitative study has found that trust and confidence in their dentist may allow implant patients to be satisfied with treatment regardless of complications [56].

### 4.2. The Proposed Standardized Questionnaire Used to Assess Patients' Self-Perceptions of Aesthetic Outcomes in Implant Dentistry

After identifying and appraising the available literature in implant dentistry used for patients assessing their own aesthetic outcomes, the authors (K. I. A., M. K. A., and S. R. B.) have proposed a standardized questionnaire that consists of a self-administered questionnaire that used a VAS, shown in (Table 3). The questionnaire included 6 items about the participants' perception of the single-tooth implant regarding overall satisfaction, comfort, tooth appearance, gum appearance, function, and cleaning complexity.

The proposed questionnaire may be used not only for single-tooth implant in the anterior zone but also for other fixed and removable prosthetic solutions assisted by dental implants. Nevertheless, the introduced questionnaire is expected to be validated for recommending it as the accepted tool to obtain PROMs from implant patients in future studies.

### 4.3. Should We Opt for Qualitative Studies in Dental Medicine to Obtain Rich “Subjective” Data?

Qualitative studies in dentistry have the following objectives that could be relevant to further assessing patient perceptions relative to their treatment: to explore different aspects of patient experience, to identify areas for improvement in patient care, and to gather information on developing strategies to increase patient satisfaction and motivation towards their oral care services and health [73].

There have been a few qualitative studies published about tooth loss effects on patients' life experiences. Broadly speaking, these studies have shown that loss of teeth is related to low functional satisfaction and reduced social confidence, as well as self-image and self-esteem concerns [74].

#### 4.3.1. Qualitative Studies in Implant Dentistry

There are only a few qualitative studies concerning patient accounts of their experiences with ISFDPs [75]. The few available studies [56, 57, 76–78] reported that ISFDP-patients have acknowledged improvement on functionality [77, 78], confidence, social life, and self-image. However, sometimes there were concerns reported regarding initial speech difficulties [56, 57], excess salivation, tongue and cheek biting, altered food taste [57], and hygiene maintenance.

It has also been concluded that there is a need to further investigate dental implant patient expectations and future satisfaction, as well as experiences among patients with single-implant crowns, particularly among young patients [75].

#### 4.3.2. Qualitative Studies Focusing on Single-Tooth Implants

The only qualitative study published on patients with single-implant crowns was focused on understanding patient experiences with immediate implants in molar sites [79]. The study concluded that clinicians must more thoroughly explain the information related to implant longevity, prosthetic aspects, cost-effectiveness, and maintenance since participants had unrealistic expectations and inadequate information. Interestingly, the participants thought that their single molar implant did not influence their appearance and self-esteem. The study encouraged conducting additional qualitative research in implant dentistry [79].

There are no qualitative studies available related to patients with single-implant crowns in the anterior zone.

#### 4.3.3. The Need for Qualitative Studies on Single-Tooth Implants in the Aesthetic Zone

A qualitative interview study is urgently required for patients with single implants in the anterior zone to explore satisfaction with their functional and aesthetic outcomes and to understand and improve the experiences of this sector of the dental population and improve communication with it. Therefore, we propose the future investigation of a range of qualitative issues related to the experiences and perceptions of the satisfaction of participants with their single-tooth implant in the maxillary anterior region.

## 5. Conclusions

  This literature review demonstrates that single-tooth anterior dental implants in the anterior are a viable treatment option, but that they can lead to some lingering challenges with the position of the implants and the soft tissue contours, in addition to some challenges with tooth color and contours, along with the potential for long-term complications and maintenance issues.
  It is encouraging to observe that there is a tendency in recent years to report PROMs more frequently in the implant dentistry literature. Moreover, the forecast of this trend prepares the scientific consumers for an expected exponential growth in the dental literature reporting PROMs to the point that it may become a requirement for future studies to be considered for publication.
  Objective and subjective indices have been developed to document and understand these issues' impact on patient satisfaction with the single-implant crown outcomes. The problem with these studies' findings is that they evidence an inconsistent and poorly explained relationship between patient satisfaction and objective indices of implant outcomes.
  PES/WES is still the most widely accepted tool to assess the aesthetic outcomes of single implant-supported crowns in the maxillary anterior region from a clinician (objective) perspective despite introducing other indices aiming to improve the inter- and intraraterreliability of such a tool.
  Several factors influence patient perspectives of implant treatment and add to the complexity of using the current nonvalidated and nonstandardized self-assessment questionnaires. Therefore, the present study introduces a unified, standardized questionnaire that consists of a self-administered questionnaire that used a VAS scoring system, which evaluates the overall satisfaction, comfort, tooth appearance, gum appearance, function, and cleaning complexity.
  As an alternative to the introduced quantitative self-administered questionnaire, conducting qualitative studies in dental implant patients who received few implants or single-tooth implant reconstructions in the aesthetic zone may help dental researchers understand better how to efficiently develop a new quantitative instrument that shall be further tested.

## Figures and Tables

**Table 1:** Criteria of the commonly used aesthetic indices and calculation of relative aesthetics.

Items	PES	PES/WES	ICAI	PICI
Criteria of the peri-implant mucosa	Mesial papilla Distal papilla Level of soft‐tissue margin Soft‐tissue contour Alveolar process deficiency Soft‐tissue color Soft‐tissue texture	Mesial papilla Distal papilla Facial curvature Level of facial mucosa Root convexity color	Labial margin Papillae Contour of the labial surface Color and surface	Papillae Zenith Root convexity

Criteria of the implant crown	N/A	Tooth form Outline/volume Color (hue/value) Surface texture Translucency and characterization	Width Length Labial convexity Color/translucency Surface	Shape Color Characterization

Subjective overall criteria	None	None	None	Crown Mucosa Overall (crown and mucosa)

Reference tooth	Contralateral tooth	Contralateral tooth	Contralateral and adjacent tooth	Contralateral tooth

Scores per criteria	2 (no deviation) 1 (small deviation) 0 (large deviation)	2 (no deviation) 1 (small deviation) 0 (large deviation)	0 (no deviation) 1 (small deviation) 5 (large deviation)	100 mm visual analogue scale

Overall score (points)	0–14	0–20	0–45	0–600

Threshold of clinical acceptability (points)	N/A	≥12	<5 P	≥360

Calculation to percentage scale	N/A	0 points = 0% 10 points = 50% 20 points = 100%	0 points = 100% 2.5 points = 50% 5 points = 0%	0 points = 0% 300 points = 50% 600 points = 100%

PES, Pink Esthetic Score; PES/WES, PES/White Esthetic Score; ICAI, Implant Crown Aesthetic Index; PICI, Peri-Implant and Crown Index. The Papilla Index (PI) assesses the size of the interproximal gingival papilla height adjacent to implant‐supported single‐tooth restorations using a score from 0 to 4: 0 = no papilla present; 1 = less than half of the papilla height is present, and a convex nature of the adjacent tissue is noted; 2 = more than half of the papilla height is present but not to the full extent of the contact point (papilla is not in complete harmony); 3 = the papilla fills the entire proximal space and is in good harmony; 4 = the papilla is hyperplastic. Thus, a complete papilla formation will achieve 3 points.

**Table 2:** Pink and White Esthetic Score sheets as interpreted by Belser et al. 2009.

*Modified Pink Esthetic Score (PES)*				
Parameter	Major discrepancy	Minor discrepancy	No discrepancy	Parameter score (minimum: 0 maximum: 2)
Mesial papilla	0	1	2	______
Distal papilla	0	1	2	______
Curvature of facial mucosa	0	1	2	______
Level of facial mucosa	0	1	2	______
Root convexity/soft tissue color and texture	0	1	2	______
Total PES score (maximum: 10)				______

*Modified White Esthetic Score (WES)*				
Parameter	Major discrepancy	Minor discrepancy	No discrepancy	Parameter score (minimum: 0 maximum: 2)

Tooth form	0	1	2	______
Tooth volume/outline	0	1	2	______
Color (hue/value)	0	1	2	______
Surface texture	0	1	2	______
Translucency	0	1	2	______
**Total PES score** (maximum: 10)				______

**Table 3:** The proposed standardized questionnaire used to subjectively assess patients' self-perceptions of aesthetic outcomes in implant dentistry.

(1) OVERALL SATISFACTION with your restored implant tooth
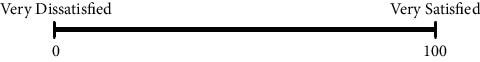
(2) OVERALL COMFORT with your implant tooth
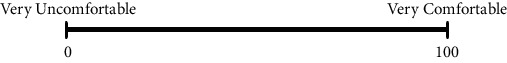
(3) Satisfaction with the APPEARANCE OF THE TOOTH (white portion) of your dental implant
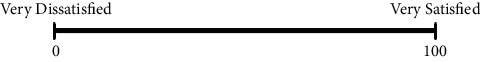
(4) Satisfaction with the APPEARANCE OF THE GUMS (pink portion) around your dental implant
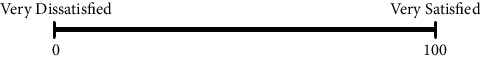
(5) OVERALL FUNCTIONING (for example, speech and chewing) with your implant tooth
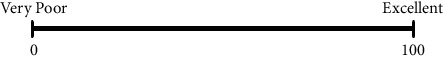
(6) CLEANING DIFFICULTY with your implant tooth